# A Few Remarks on Cholera

**Published:** 1866-08

**Authors:** T. F. Rochester


BUFFALO

### Medical and Surgical Journal.

VOL. VI. AUGUST, 1866. No. 1.

### Original Communications.

### ART. I—*A few Remarks on Cholera.* By T. F. Rochester, M. D.

(CONCLUDED.)

In the preceding part of this article reference was made to the mischievous doctrine of George Johnson, M. D., of London, to the effect that vomiting and purging were sanitary efforts on the part of nature, to eliminate the choleraic poison. As a natural sequence of such a notion, the administration of cathartics is advocated—and castor oil is specially selected—from the mildness and efficiency of its action. Dr. Johnson claims priority in this treatment, dating from 1854. The writer has repeatedly seen not only castor oil, but even croton oil, given in 1849, under the direction of the late Dr. Vaché, who had charge of a cholera hospital near Chatham Square, in the city of New York. He was a talented, eccentric man, but not a judicious practitioner, and held similar views to Dr. Johnson. The writer also remembers what was aptly called "the Castor Oil Mania" in London in 1854. It was recommended in a public journal, by some prominent practitioner, (probably Dr. Johnson,) not only as a curative, but as a preventive. Everybody, as the phrase goes, took castor oil. The number of cases of cholera increased correspondingly, and were so directly traceable to the use of the oil, that placards were posted in various parts of the city, warning people of the danger of its employment. Dr. Johnson says, "there is no specific against cholera, and probably never will be." To this remark, unhappily, we are obliged to assent. Admitting that there is no specific, what are the rational indications for treatment? These would seem to be, *First*, to arrest the serous discharges, and thus prevent collapse. *Second*, to sustain the strength of the patient. *Third*, to encourage the return of the urinary secretion, and thus avoid uræmia. *Fourth*, to guard against cerebral engorgement and secondary fever. It is not within the scope or object of this paper to discuss preventive or hygienic measures, and therefore only insisting upon their extreme importance, we pass at once to the consideration of the *treatment* of cholera, and in doing this, we take the ground that, cholera prevailing, *painless diarrhœa* is its first stage, subject to the exceptions before noted. The evacuations may or may not be serous, and may or may not present the rice water appearance. The disease in this stage is usually easily controlled by simple treatment. It is all important that the recumbent position should be maintained, that the inclination to vomit or to evacuate the bowels should be resisted, that the surface of the body should be kept covered sufficiently to maintain warmth, but not to such an extent as to be oppressive. Very little food should be taken, and that of very simple character, and this should be *drg* rather than fluid. Tea and toast, gruels and dilute animal or vegetable broths should be eschewed. This is indeed properly called "sick diet," but not in the sense generally understood. As to medicine, opium alone, or in combination, is probably the most efficient agent, but opium should not be used in large doses or be very frequently repeated in small. Hence its combination with chalk, or with camphor or capsicum, or some other antacid or stomachic is generally with good reason preferred. Mercury is also very useful, but of course should not be given, if other and more simple drugs will suffice. It may be used with or without opium. It is often wonderfully efficient in a very mild form, and in a very moderate amount, as the writer has repeatedly observed, and notably at the Alms-house on Blackwell's Island in 1849, of which institution he had the medical charge when the cholera broke out among its inmates. One of two blue pills of five grains each, without any other medication, entirely controlled the premonitory diarrhœa, in almost every instance where early attention was called to it; and in a class of patients whose condition and hygienic surroundings were, like that of most paupers, as bad as possible. By this statement, it is not intended to be understood that blue pill will always arrest choleraic diarrhœa, but that it will often to do. It would be very unwise to advise patients to take it, at their own discretion, for this purpose, but it is doubtful if it would be more productive of harm than the various "cholera drops" that are found in almost every household; for these combinations of laudanum, camphor, capsicum, chloroform, rheubarb, peppermint, etc., are often resorted to from apprehension alone, and the digestion is so much deranged thereby, that diarrhœa, which only existed in the imagination, not unfrequently ensues. It often happens that the premonitory diarrhœa although arrested for a few days, returns, either spontaneously, or through some indiscretion in diet, or from over fatigue. A flannel bandage fastened tightly around the abdomen and worn constantly will often prevent this recurrence. Those who are much exposed to the night air and those who sleep habitually on the ground floor are much more subject to diarrhœal attacks than those who remain indoors, and who occupy the second or third stories. In those cases where a decided proneness to diarrhœa continues, nothing is as effectual as a change of air, to the sea coast, or to a high and dry country region, but a person, ill with diarrhœa, should never leave home and his usual medical attendant until the disease is, for the time, at least, entirely arrested. Many from neglecting this precaution, have been seized and have died en-route with fully developed cholera. And this brings us to the most important part of our subject—the treatment of impending or established cholera collapse, with the rice water evacuations. It is that alarming stage of the disease, over which medicine has in many instances but little control, as all honest practitioners, who have seen much of the disorder, concur in saying; but although true in many instances, happily the statement does not apply to all. Of this, the writer had an opportunity of observing a very good illustration in Algiers in 1850. The natives, like all Mahomedans, are fatalists, and took no measures to stay the progress of the disease. The mortality among them was much greater than among the foreign residents, who sought the aid of the resident French physicians. Serous or rice water evacuations occurring, how are they to be arrested? To this it may be replied, that almost every drug and appliance, at all likely to accomplish this end had been tried, and that articles and methods highly praised by some practitioners, have entirely failed in the hands of others. This is to be accounted for by the different type of the disease in various places and by essential variation in the disease itself in the same place in its recurrence after an interval of one or more years, and also and not less important, by the difference in the judgment and assiduity of practitioners themselves. If the writer indicates a plan of treatment to be generally followed, he does so in no spirit of egotism, and with all respect to the opinion of those entertaining different views. It is the result of extended personal observation and experience, he has tried and has seen tried very many methods of treatment, and while he candidly admits, that nothing is very reliable or satisfactory, his decided preference is as follows: By all means secure for the patient the largest and best ventilated room in the house, one on the second or third story, and with an open fire-place, if possible; order immediately strong beef essence to be prepared, and send for ice. Place the patient's bed in the middle of the room, and let him be placed between blankets. Insist upon his keeping the surface of the body covered, and by no means allow him to rise either to vomit or to evacuate the bowels; basins and bed-pans can be so arranged as to prevent this necessity. Give at one dose, of calomel, opium and capsicum, each two grains, either in pill or powder. This is best administered immediately after vomiting. If the patient is very thirsty or is inclined to vomit, let him swallow small pieces of ice, but cause him to abstain, for a time at least, from fluids of every description. Dry cups over the epigastrium will materially assist in preventing gastric regurgitation. An enema of from one to two drachms of laudanum in a small amount of starch water, should immediately succeed an alvine dejection. The object of the treatment thus far is to make a quick and decided *opium effect* upon the system, with the hope of thus doing much toward the arrest of the discharges, and also to gain time for other agents to accomplish more permanently the end. The capsicum is given not only as a valuable stimulant and astringent, but also by its local action upon the gastric mucous membrane, to promote the absorption of the opium, and thus prevent the justly dreaded cumulative effect of repeated doses of the narcotic. The writer has no experience of hypodermic medication in cholera, but hopes that morphine, thus used, will have an approximation at least, to its rapid and happy effect in cholera morbus. The calomel, in the triple combination, is given, not as Dr. Johnson terms it, as a cathartic, nor as an *alterative*, as it is often vaguely and mischievously called, but as a *sedative* to the extreme action of the mucous membrane of the stomach and bowels; that it does possess this property, we have good reason to believe from its indisputable effect in cholera infantum and some other disorders; and it is from belief in this action, that in the further prosecution of the treatment of cholera, we advise that one grain of calomel be laid upon the tongue with a little pounded ice, every half hour, for from six to twelve hours, and then at longer and longer intervals. The opium, calomel and capsicum pills should be repeated every two to four hours, according to the evidences of narcotic effect. If the pupil is contracted, and if the respiration becomes slower or unequal, opium should be withheld. It will be remembered that on post mortem examinations the gall bladder is usually found filled with the biliary secretion. Calomel need not therefore be administered as a cholagogue, but as the rice water ceases to be secreted the duodenal orifice of the common duct is no longer as it were pasted up, and bile is seen in the stools and in the discharges from the stomach. It is rather an evidence of amendment than the cause of it. It was formerly supposed that when greenish stools appeared, the patient was safe; this has proved to be an error. It has been urged that calomel should not be given in the manner advocated, on account of the great liability of salivation. It will salivate but very few; but if it is potential in permanently arresting the "serous diarrhœa," and also in arresting some of the choleraic sequelæ, salivation is of minor importance.

The nutrition of the patient next demands attention; this is a vital point too apt to be overlooked. For this purpose we require food, small in bulk and easily assimilated. Beef essence or concentrated mutton broth should be given frequently, but in very limited amount, as soon as the stomach can possibly retain it, even if a portion is often thrown off. The idea that food is not digested or assimilated in collapse, is certainly to a considerable extent erroneous. One would naturally also suppose that stimulants (alcoholic) should be freely used, but this is by no means certain. They are sometimes beneficial, oftener useless, and occasionally apparently hurtful. The writer thinks he has employed iced champagne, however, with great advantage; it has often appeared to arrest vomiting, to sustain the patient, and to restore the renal secretion. Its diuretic property is certainly an element in its favor. Soda water with gin, whisky or brandy, is less efficient, but makes the nearest approach to it that is available to the poorer classes. It will be remembered that abstinence from liquids, for a time, has been advised—for how long a time is a question difficult to answer. The urgent thirst, and the waste of fluid demand respectively appeasement and supply; but so often are the vomiting and purging immediately renewed, seemingly from the act of swallowing fluids, and especially water, that one's judgment is often at variance with the kindly feeling that urges temporary relief. In the terrible outbreak of cholera at Suspension Bridge in 1854, the writer suggested to Dr. Hunt and other physicians, who nobly volunteered to go there, that water should be given in unlimited quantities, and that it should also be applied freely to the surface by means of the wet sheet. The effect was to relieve both cramps and thirst in the only two instances in which it was tried, and one of these recovered. It was employed just at the close of Dr. Hunt's service, and he was favorably impressed with its action. The most painful phenomenon of cholera is cramp. This is abated by friction, but it is probable that the process of rubbing exhausts the muscular power of the patient, and also directs the current of blood toward the trunk. If recourse is had to it, it should therefore be made toward the extremities. The late Prof. William E. Horner of Philadelphia, thought that chloroform administered in ten drop doses, every hour, allayed and prevented cramp. Many others entertain the same opinion. Camphor is also supposed to be very efficacious in relieving not only this but other choleraic complications. Its use is highly commended by many physicians. A homœpathist in this city claims to have treated over one hundred cases successfully, (losing only one patient,) with one drop of the saturated tincture, given every five minutes. This is in no respect a homœpathic dose, but the statement is of no value, and is like all similar ones emanating from what source they may, worthless, from the fact that too much is claimed, and from the necessary conclusion, that the patients thus treated, did not have cholera. The writer is of the opinion that camphor is of but little value, except as a stimulant and antispasmodic, but that it may be advantageously employed in connection with other agents. One great feature in the treatment of cholera, is to give something every few minutes, that will counteract the continual tendency to sinking; possibly this property may exist in camphor, and it is suggested that a drop or two of the saturated solution in chloroform, frequently given, may be worth trying.

To maintain, or to cause to return, when suspended, the urinary secretion, is of the first importance. This is to be encouraged by keeping the lumbar and abdominal regions swathed in sheets wet with warm water, and by the subsequent administration of mild diuretics. Injections of warm water into the urinary bladder are said to stimulate the kidneys into action, and may be useful as affording a depot of water to be absorbed into the system, although ordinarily no such absorption would take place.

We now pass to the fourth indication for treatment, viz: To guard against cerebral engorgement and secondary fever. This is a condition which does not belong to all cases of cholera, but is rather peculiar to certain epidemics of it. In 1832, it was scarcely encountered. It was met with frequently in 1849. There was little of it in 1852, while in 1854 it was very common. It is not a simple excessive reaction. Neither is it the result of the free administration of opium, for it is often met with where no opium has been taken, and it is often absent where opium has been taken very largely and continuously. It is a sequel or complication full of danger, and often snatches away the fairest prospects of recovery. It is not a typhoid state, but one of stupor without delirium, and pathologically, a passive engorgement of the brain and its membranes with serous exudation into the cranial and spinal cavities. This condition, we repeat, is not the result of the use of opium, as has been alleged, but the epidemic predisposition exististing; opium may, if recklessly employed, increase it, and certainly should not be administered when there are manifestations of its incipiency. It is probably best treated by shaving the scalp, by the application of the ice cap, by keeping the head elevated, by the administration of the iodide or bromide of potash, and by a regimen of beef essence. Cups, leeches and blisters to the nuchæ and temples are also useful, but of secondary importance. *Here*, with deep coma, a derivative cathartic, even croton oil may do good, but as a greenish diarrhœa and an irritable stomach often co-exist with the cerebral symptoms, cathartics are of questionable utility.

The late and lamented Dr. I. M. Newman of this city, in a very able paper read before the Buffalo Medical Association October 3d, 1854, said, "Before concluding this article, I wish to say a few words upon the therapeutical effects of cold, by the means of ice or water, applied to the head in cholera, and to urge it upon the profession as a remedy not undeserving trial. Its application will undoubtedly be noticed, as among the remedial measures used in the cases of congestion of the brain which I have just related. From employing it in cases of brain complications only, I gradually extended the sphere of its application, until it embraced cases of every stage, from the first seizure to that of complete collapse, and from the observation of its effects in a large number of cases, I have no hesitation in saying, that it is among the most valuable means we possess of controlling the vomiting of cholera. Its influence in some cases was almost magical, quieting the action of the stomach, and enabling the patient to take and retain medicine and drinks." He concludes as follows: "I have spoken of congestion of the brain as a *stage* of cholera, * * * * * * I am now prepared to advance a step farther, and claim, that the first manifestations of the specific cause of cholera, upon the human organism, be that cause what it may, is *upon the brain*, and through it *upon the nervous centers*. * * * * * If this view be correct we can understand how the application of cold may be made to assume a prominent rank among our medicines." Whether Dr. Newman was right or wrong in this idea, it will be seen that he anticipated Dr. John Chapman of ice and hot-water-bag notoriety, in what he terms his theory (Chapman's) of cholera and its treatment.

In bringing this article to a close, the writer asks the indulgence of the reader. It has necessarily been written at odd moments, and those subject to constant interruptions. It will be observed that he has said nothing about the thousand and one articles that are claimed as more or less specific by persons in and out of the profession. It is because he has used or has seen used many of them, and has found them all more or less failures; and while he does not claim its great success, but rather deplores its frequent inutility, he presents the method of treatment described above, as that in which he has the most faith, and with which he has had a fair share of success.

